# Cerebellar hemorrhage with germinal matrix intraventricular hemorrhage: Continuum from fetal to neonatal life

**DOI:** 10.1111/dmcn.16176

**Published:** 2024-11-07

**Authors:** Rony Cohen, Tally Lerman‐Sagie

**Affiliations:** ^1^ Fetal Brain Research Center Wolfson Medical Center Holon Israel

## Abstract

This commentary is on the original article by Buchmayer et al. on pages 609–617 of this issue.

Cerebellar hemorrhage has been associated with germinal matrix intraventricular hemorrhage (GM‐IVH), particularly in extremely low birthweight (ELBW) infants born preterm before 28 weeks of gestation.^1^ This condition may arise due to blood spillage into the fourth ventricle in cases of high‐grade IVH or the simultaneous occurrence of cerebellar hemorrhage. Although the exact pathogenesis remains unclear, it has been suggested that both conditions share a common mechanism.^2^ The rapid angiogenesis in the cerebellar germinal matrix between 20 weeks and 30 weeks of gestation leads to the development of immature blood vessels, making the cerebellum more vulnerable to hemorrhage.

Buchmayer et al.^3^ provide valuable insights into the relationship between GM‐IVH and cerebellar hemorrhage in infants born preterm. They demonstrate that 70% of infants born extremely preterm with GM‐IVH also experience cerebellar hemorrhage, and one‐third consequently develop cerebellar atrophy.

The study highlights that the association of GM‐IVH and cerebellar hemorrhage causes significantly higher rates of cerebral palsy and cognitive impairment, whereas cerebellar atrophy is even more strongly linked to generally worse neurodevelopmental outcome. Although this link with more adverse prognosis is well documented in the paper, it is crucial to demonstrate in further studies the full range of neurodevelopmental difficulties by performing a thorough neurological evaluation by a paediatric neurologist. The use of the Scale for the Assessment and Rating of Ataxia is particularly important for evaluating motor function and ataxia in children with cerebellar disruptive anomalies. In contrast, the Gross Motor Function Classification System is less effective at distinguishing spasticity and ataxia in non‐ambulatory children, potentially leading to inaccurate conclusions.

Some of the pathogenic factors for GM‐IVH and cerebellar hemorrhage elucidated in infants born extremely preterm also exist in fetuses at the same gestational age. However, in a large systemic review and meta‐analysis on antenatal IVH,^4^ this association has not been described. We have recently encountered this combination in a cohort of fetuses with IVH,^5^ and believe it is actually not so rare.

We propose that to gain a deeper understanding of cerebellar hemorrhage in the context of GM‐IVH, it is valuable to explore its occurrence and pathogenesis during fetal life. The risk factors in utero differ, as the immature brain is not yet exposed to the systemic hemodynamic stressors brought on by preterm birth and the extrauterine environment.

Recent advances in prenatal neurosonography and the increased use of brain MRI have contributed to a rise in the prenatal diagnosis of GM‐IVH. However, the association between GM‐IVH and cerebellar hemorrhage in fetuses has been scarcely documented despite the frequent association between cerebellar bleeding and unilateral cerebellar hypoplasia. The aetiology of isolated antenatal cerebellar hemorrhage has been attributed to maternal factors, such as trauma, placental insufficiency, seizures, and certain medications, or fetal factors, including vascular malformations, congenital infections, and thrombocytopenia and not to concurrent GM‐IVH.

We suggest that an international multicenter study on fetal GM‐IVH with cerebellar hemorrhage and postnatal outcome would complement the important study by Buchmayer et al.^3^ Such a study should incorporate the new parameters presented by Hadi et al.^5^ for the evaluation of fetal IVH, including the involvement of the posterior fossa and focusing on imaging patterns, underlying aetiologies, and predictors that influence clinical outcome.

## Data Availability

Not required.

## References

[1] Limperopoulos C , Benson CB , Bassan H , Disalvo DN , Kinnamon DD , Moore M , et al. Cerebellar hemorrhage in the preterm infant: ultrasonographic findings and risk factors. Pediatrics. 2005; 116: 717–24. 16140713 10.1542/peds.2005-0556

[2] Volpe JJ . Cerebellum of the premature infant: rapidly developing, vulnerable, clinically important. J Child Neurol. 2009; 24: 1085–104. 19745085 10.1177/0883073809338067 PMC2799249

[3] Buchmayer J , Fuiko R , Kienast P , Stummer S , Kasprian G , Berger A , et al. Cerebellar haemorrhage and atrophy in infants born extremely preterm with intraventricular haemorrhage. Dev Med Child Neurol. 2024 Oct 20. 10.1111/dmcn.16123. Online ahead of print. PMC11965970 39428664

[4] Dunbar MJ , Woodward K , Leijser LM , Kirton A . Antenatal diagnosis of fetal intraventricular hemorrhage: systematic review and meta‐analysis. Dev Med Child Neurol. 2021; 63: 144–55. 33094492 10.1111/dmcn.14713

[5] Hadi E , Haddad L , Levy M , Gindes L , Hausman‐Kedem M , Bassan H , et al. Fetal intraventricular hemorrhage and periventricular hemorrhagic venous infarction: time for dedicated classification system. Ultrasound Obstet Gynecol. 2024; 64: 285–93. 38363592 10.1002/uog.27613

